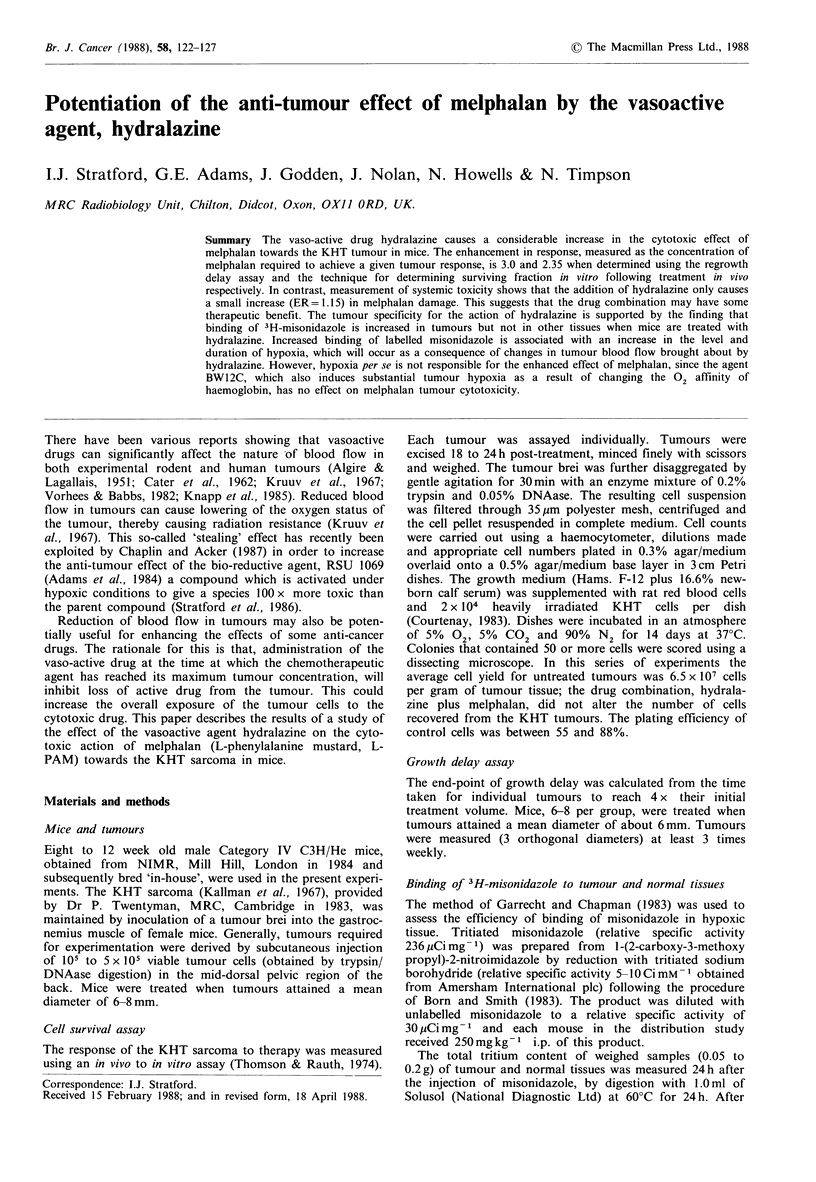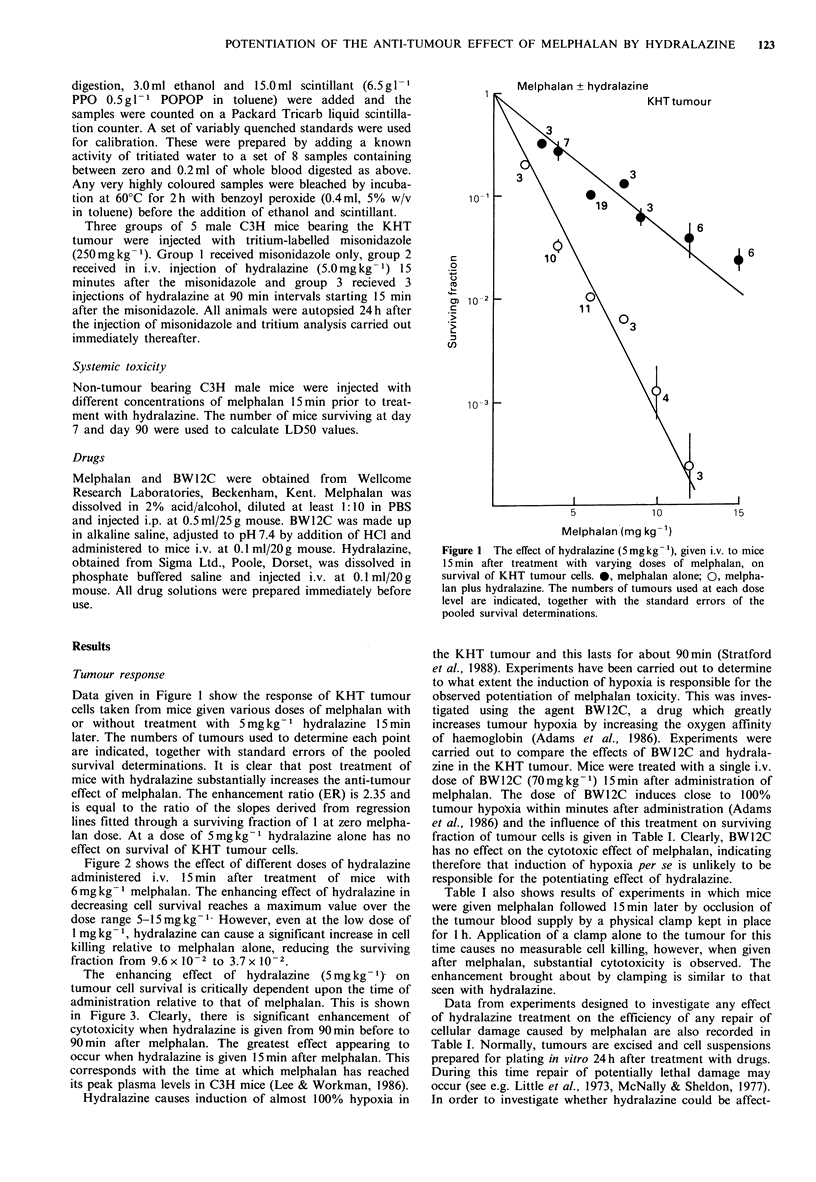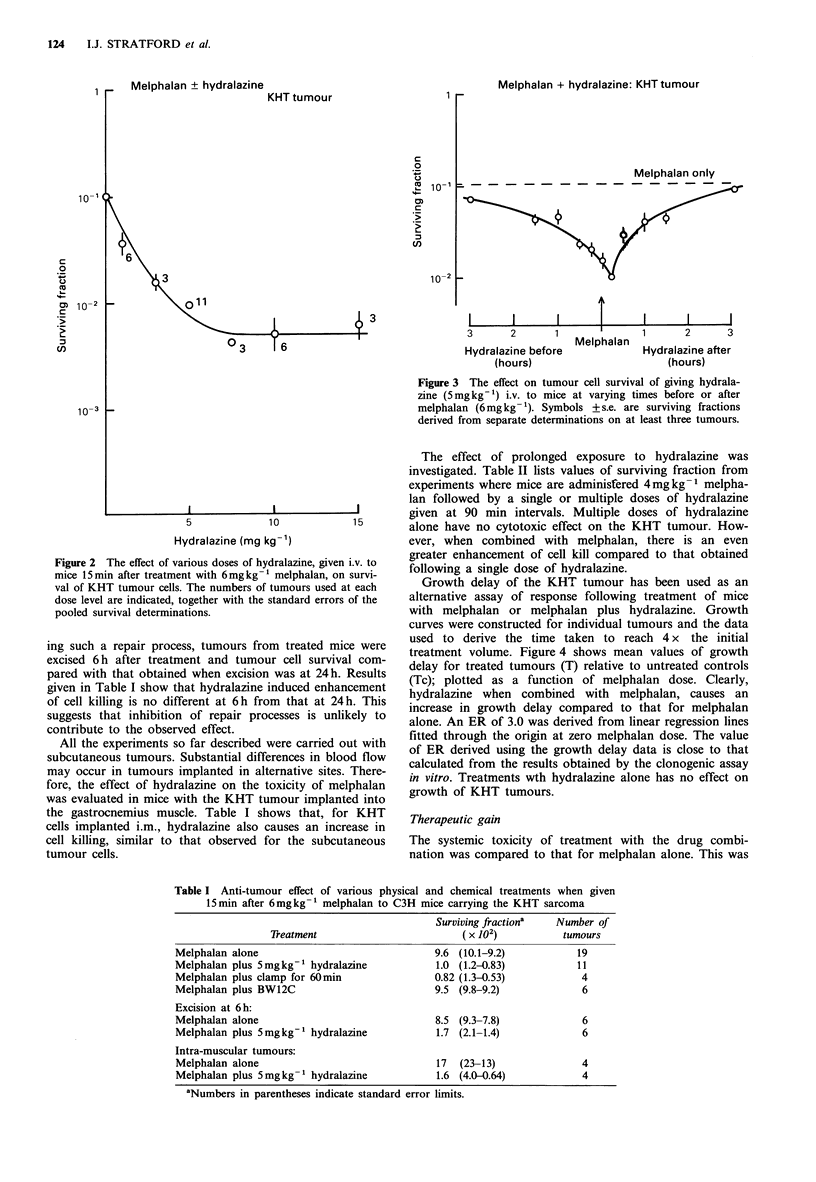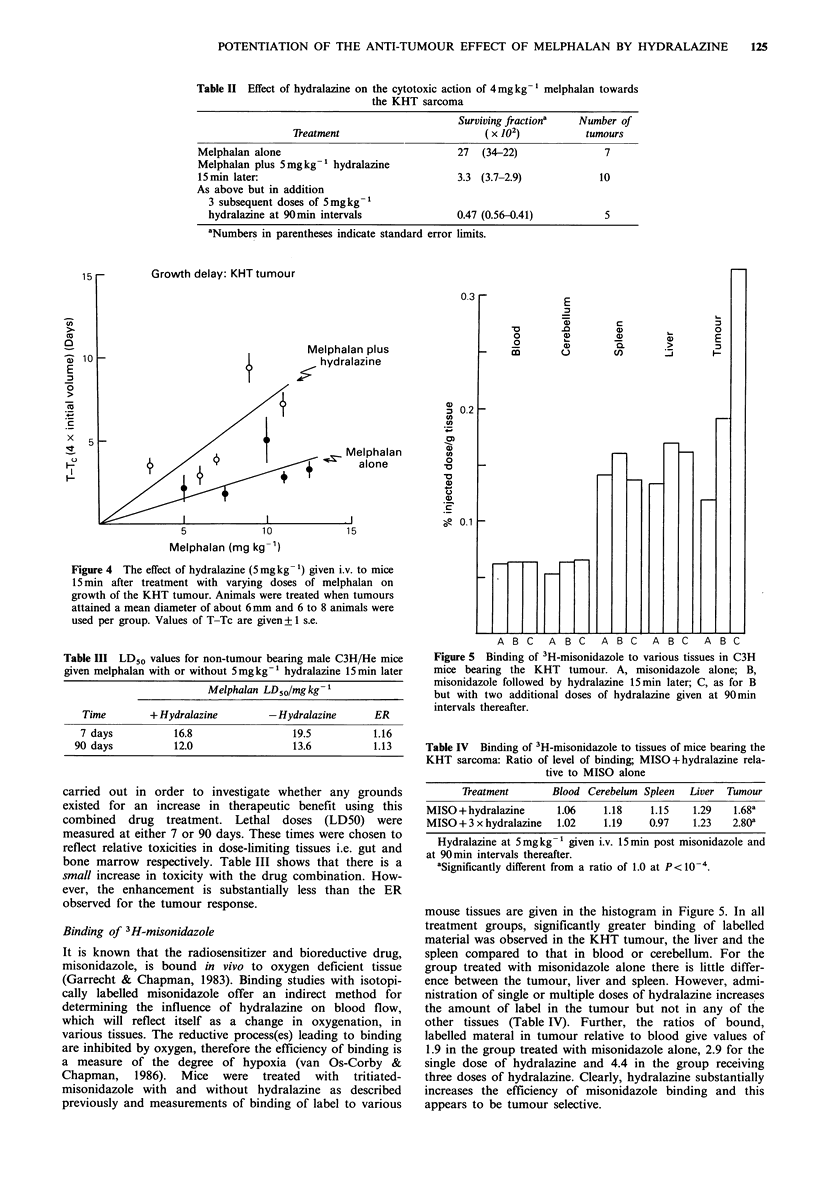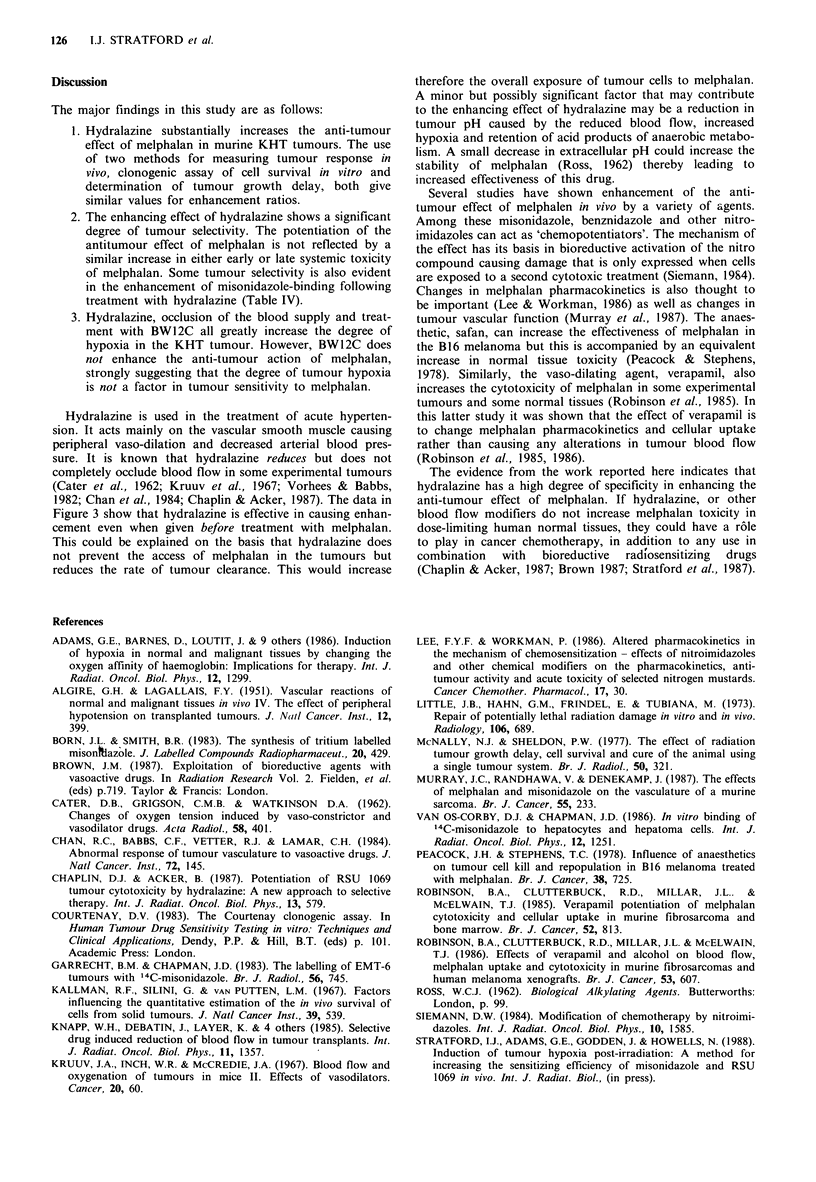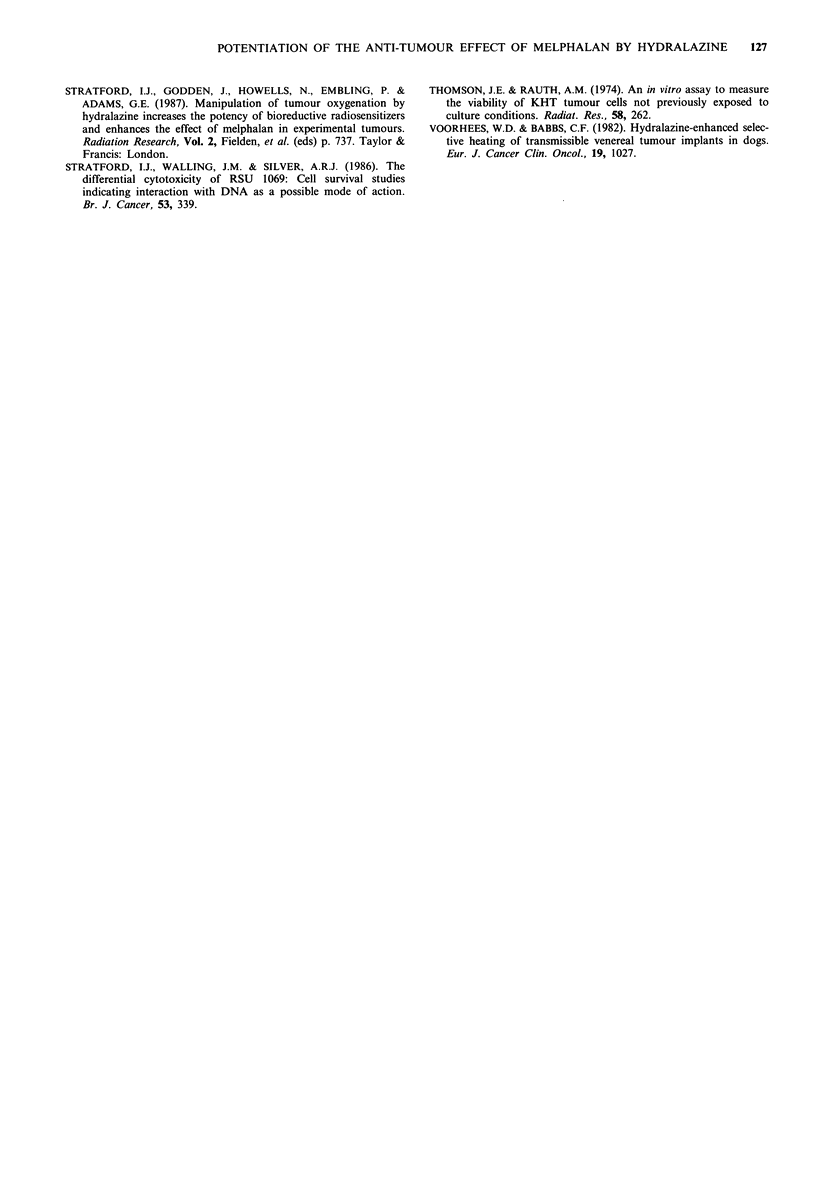# Potentiation of the anti-tumour effect of melphalan by the vasoactive agent, hydralazine.

**DOI:** 10.1038/bjc.1988.177

**Published:** 1988-08

**Authors:** I. J. Stratford, G. E. Adams, J. Godden, J. Nolan, N. Howells, N. Timpson

**Affiliations:** MRC Radiobiology Unit, Oxon, UK.

## Abstract

The vaso-active drug hydralazine causes a considerable increase in the cytotoxic effect of melphalan towards the KHT tumour in mice. The enhancement in response, measured as the concentration of melphalan required to achieve a given tumour response, is 3.0 and 2.35 when determined using the regrowth delay assay and the technique for determining surviving fraction in vitro following treatment in vivo respectively. In contrast, measurement of systemic toxicity shows that the addition of hydralazine only causes a small increase (ER = 1.15) in melphalan damage. This suggests that the drug combination may have some therapeutic benefit. The tumour specificity for the action of hydralazine is supported by the finding that binding of 3H-misonidazole is increased in tumours but not in other tissues when mice are treated with hydralazine. Increased binding of labelled misonidazole is associated with an increase in the level and duration of hypoxia, which will occur as a consequence of changes in tumour blood flow brought about by hydralazine. However, hypoxia per se is not responsible for the enhanced effect of melphalan, since the agent BW12C, which also induces substantial tumour hypoxia as a result of changing the O2 affinity of haemoglobin, has no effect on melphalan tumour cytotoxicity.


*Br. J. Cancer* (1988), **58**, 122–127 © The Macmillan Press Ltd., 1988

### Potentiation of the anti-tumour effect of melphalan by the vasoactive agent, hydralazine

I.J. Stratford, G.E. Adams, J. Godden, J. Nolan, N. Howells & N. Timpson

*MRC Radiobiology Unit, Chilton, Didcot, Oxon, OX11 0RD, UK.*

**Summary** The vaso-active drug hydralazine causes a considerable increase in the cytotoxic effect of melphalan towards the KHT tumour in mice. The enhancement in response, measured as the concentration of melphalan required to achieve a given tumour response, is 3.0 and 2.35 when determined using the regrowth delay assay and the technique for determining surviving fraction *in vitro* following treatment *in vivo* respectively. In contrast, measurement of systemic toxicity shows that the addition of hydralazine only causes a small increase (ER = 1.15) in melphalan damage. This suggests that the drug combination may have some therapeutic benefit. The tumour specificity for the action of hydralazine is supported by the finding that binding of $^3$H-misonidazole is increased in tumours but not in other tissues when mice are treated with hydralazine. Increased binding of labelled misonidazole is associated with an increase in the level and duration of hypoxia, which will occur as a consequence of changes in tumour blood flow brought about by hydralazine. However, hypoxia *per se* is not responsible for the enhanced effect of melphalan, since the agent BW12C, which also induces substantial tumour hypoxia as a result of changing the $O_2$ affinity of haemoglobin, has no effect on melphalan tumour cytotoxicity.

There have been various reports showing that vasoactive drugs can significantly affect the nature of blood flow in both experimental rodent and human tumours (Algire & Lagallais, 1951; Cater *et al.*, 1962; Kruuv *et al.*, 1967; Vorhees & Babbs, 1982; Knapp *et al.*, 1985). Reduced blood flow in tumours can cause lowering of the oxygen status of the tumour, thereby causing radiation resistance (Kruuv *et al.*, 1967). This so-called 'stealing' effect has recently been exploited by Chaplin and Acker (1987) in order to increase the anti-tumour effect of the bio-reductive agent, RSU 1069 (Adams *et al.*, 1984) a compound which is activated under hypoxic conditions to give a species 100× more toxic than the parent compound (Stratford *et al.*, 1986).

Reduction of blood flow in tumours may also be potentially useful for enhancing the effects of some anti-cancer drugs. The rationale for this is that, administration of the vaso-active drug at the time at which the chemotherapeutic agent has reached its maximum tumour concentration, will inhibit loss of active drug from the tumour. This could increase the overall exposure of the tumour cells to the cytotoxic drug. This paper describes the results of a study of the effect of the vasoactive agent hydralazine on the cytotoxic action of melphalan (L-phenylalanine mustard, L-PAM) towards the KHT sarcoma in mice.

### Materials and methods

### *Mice and tumours*

Eight to 12 week old male Category IV C3H/He mice, obtained from NIMR, Mill Hill, London in 1984 and subsequently bred 'in-house', were used in the present experiments. The KHT sarcoma (Kallman *et al.*, 1967), provided by Dr P. Twentyman, MRC, Cambridge in 1983, was maintained by inoculation of a tumour brei into the gastrocnemius muscle of female mice. Generally, tumours required for experimentation were derived by subcutaneous injection of $10^5$ to $5\times10^5$ viable tumour cells (obtained by trypsin/DNAase digestion) in the mid-dorsal pelvic region of the back. Mice were treated when tumours attained a mean diameter of 6–8 mm.

### *Cell survival assay*

The response of the KHT sarcoma to therapy was measured using an *in vivo* to *in vitro* assay (Thomson & Rauth, 1974). Each tumour was assayed individually. Tumours were excised 18 to 24 h post-treatment, minced finely with scissors and weighed. The tumour brei was further disaggregated by gentle agitation for 30 min with an enzyme mixture of 0.2% trypsin and 0.05% DNAase. The resulting cell suspension was filtered through 35 $\mu$m polyester mesh, centrifuged and the cell pellet resuspended in complete medium. Cell counts were carried out using a haemocytometer, dilutions made and appropriate cell numbers plated in 0.3% agar/medium overlaid onto a 0.5% agar/medium base layer in 3 cm Petri dishes. The growth medium (Hams. F-12 plus 16.6% new-born calf serum) was supplemented with rat red blood cells and $2\times10^4$ heavily irradiated KHT cells per dish (Courtenay, 1983). Dishes were incubated in an atmosphere of 5% $O_2$, 5% $CO_2$ and 90% $N_2$ for 14 days at 37°C. Colonies that contained 50 or more cells were scored using a dissecting microscope. In this series of experiments the average cell yield for untreated tumours was $6.5\times10^7$ cells per gram of tumour tissue; the drug combination, hydralazine plus melphalan, did not alter the number of cells recovered from the KHT tumours. The plating efficiency of control cells was between 55 and 88%.

Correspondence: I.J. Stratford.
Received 15 February 1988; and in revised form, 18 April 1988.

### *Growth delay assay*

The end-point of growth delay was calculated from the time taken for individual tumours to reach 4× their initial treatment volume. Mice, 6–8 per group, were treated when tumours attained a mean diameter of about 6 mm. Tumours were measured (3 orthogonal diameters) at least 3 times weekly.

### *Binding of $^3$H-misonidazole to tumour and normal tissues*

The method of Garrecht and Chapman (1983) was used to assess the efficiency of binding of misonidazole in hypoxic tissue. Tritiated misonidazole (relative specific activity 236 $\mu$Ci mg$^{-1}$) was prepared from 1-(2-carboxy-3-methoxy propyl)-2-nitroimidazole by reduction with tritiated sodium borohydride (relative specific activity 5–10 Ci mM$^{-1}$ obtained from Amersham International plc) following the procedure of Born and Smith (1983). The product was diluted with unlabelled misonidazole to a relative specific activity of 30 $\mu$Ci mg$^{-1}$ and each mouse in the distribution study received 250 mg kg$^{-1}$ i.p. of this product.

The total tritium content of weighed samples (0.05 to 0.2 g) of tumour and normal tissues was measured 24 h after the injection of misonidazole, by digestion with 1.0 ml of Solusol (National Diagnostic Ltd) at 60°C for 24 h. After digestion, 3.0 ml ethanol and 15.0 ml scintillant (6.5 g $l^{-1}$ PPO 0.5 g $l^{-1}$ POPOP in toluene) were added and the samples were counted on a Packard Tricarb liquid scintillation counter. A set of variably quenched standards were used for calibration. These were prepared by adding a known activity of tritiated water to a set of 8 samples containing between zero and 0.2 ml of whole blood digested as above. Any very highly coloured samples were bleached by incubation at 60°C for 2 h with benzoyl peroxide (0.4 ml, 5% w/v in toluene) before the addition of ethanol and scintillant.

Three groups of 5 male C3H mice bearing the KHT tumour were injected with tritium-labelled misonidazole (250 mg $kg^{-1}$). Group 1 received misonidazole only, group 2 received in i.v. injection of hydralazine (5.0 mg $kg^{-1}$) 15 minutes after the misonidazole and group 3 recieved 3 injections of hydralazine at 90 min intervals starting 15 min after the misonidazole. All animals were autopsied 24 h after the injection of misonidazole and tritium analysis carried out immediately thereafter.

*Systemic toxicity*

Non-tumour bearing C3H male mice were injected with different concentrations of melphalan 15 min prior to treatment with hydralazine. The number of mice surviving at day 7 and day 90 were used to calculate LD50 values.

*Drugs*

Melphalan and BW12C were obtained from Wellcome Research Laboratories, Beckenham, Kent. Melphalan was dissolved in 2% acid/alcohol, diluted at least 1:10 in PBS and injected i.p. at 0.5 ml/25 g mouse. BW12C was made up in alkaline saline, adjusted to pH 7.4 by addition of HCl and administered to mice i.v. at 0.1 ml/20 g mouse. Hydralazine, obtained from Sigma Ltd., Poole, Dorset, was dissolved in phosphate buffered saline and injected i.v. at 0.1 ml/20 g mouse. All drug solutions were prepared immediately before use.

**Figure 1** The effect of hydralazine (5 mg $kg^{-1}$), given i.v. to mice 15 min after treatment with varying doses of melphalan, on survival of KHT tumour cells. ●, melphalan alone; ○, melphalan plus hydralazine. The numbers of tumours used at each dose level are indicated, together with the standard errors of the pooled survival determinations.

### Results

*Tumour response*

Data given in Figure 1 show the response of KHT tumour cells taken from mice given various doses of melphalan with or without treatment with 5 mg $kg^{-1}$ hydralazine 15 min later. The numbers of tumours used to determine each point are indicated, together with standard errors of the pooled survival determinations. It is clear that post treatment of mice with hydralazine substantially increases the anti-tumour effect of melphalan. The enhancement ratio (ER) is 2.35 and is equal to the ratio of the slopes derived from regression lines fitted through a surviving fraction of 1 at zero melphalan dose. At a dose of 5 mg $kg^{-1}$ hydralazine alone has no effect on survival of KHT tumour cells.

Figure 2 shows the effect of different doses of hydralazine administered i.v. 15 min after treatment of mice with 6 mg $kg^{-1}$ melphalan. The enhancing effect of hydralazine in decreasing cell survival reaches a maximum value over the dose range 5–15 mg $kg^{-1}$. However, even at the low dose of 1 mg $kg^{-1}$, hydralazine can cause a significant increase in cell killing relative to melphalan alone, reducing the surviving fraction from $9.6 \times 10^{-2}$ to $3.7 \times 10^{-2}$.

The enhancing effect of hydralazine (5 mg $kg^{-1}$) on tumour cell survival is critically dependent upon the time of administration relative to that of melphalan. This is shown in Figure 3. Clearly, there is significant enhancement of cytotoxicity when hydralazine is given from 90 min before to 90 min after melphalan. The greatest effect appearing to occur when hydralazine is given 15 min after melphalan. This corresponds with the time at which melphalan has reached its peak plasma levels in C3H mice (Lee & Workman, 1986).

Hydralazine causes induction of almost 100% hypoxia in the KHT tumour and this lasts for about 90 min (Stratford *et al.*, 1988). Experiments have been carried out to determine to what extent the induction of hypoxia is responsible for the observed potentiation of melphalan toxicity. This was investigated using the agent BW12C, a drug which greatly increases tumour hypoxia by increasing the oxygen affinity of haemoglobin (Adams *et al.*, 1986). Experiments were carried out to compare the effects of BW12C and hydralazine in the KHT tumour. Mice were treated with a single i.v. dose of BW12C (70 mg $kg^{-1}$) 15 min after administration of melphalan. The dose of BW12C induces close to 100% tumour hypoxia within minutes after administration (Adams *et al.*, 1986) and the influence of this treatment on surviving fraction of tumour cells is given in Table I. Clearly, BW12C has no effect on the cytotoxic effect of melphalan, indicating therefore that induction of hypoxia *per se* is unlikely to be responsible for the potentiating effect of hydralazine.

Table I also shows results of experiments in which mice were given melphalan followed 15 min later by occlusion of the tumour blood supply by a physical clamp kept in place for 1 h. Application of a clamp alone to the tumour for this time causes no measurable cell killing, however, when given after melphalan, substantial cytotoxicity is observed. The enhancement brought about by clamping is similar to that seen with hydralazine.

Data from experiments designed to investigate any effect of hydralazine treatment on the efficiency of any repair of cellular damage caused by melphalan are also recorded in Table I. Normally, tumours are excised and cell suspensions prepared for plating *in vitro* 24 h after treatment with drugs. During this time repair of potentially lethal damage may occur (see e.g. Little *et al.*, 1973, McNally & Sheldon, 1977). In order to investigate whether hydralazine could be affect-

**Figure 2** The effect of various doses of hydralazine, given i.v. to mice 15 min after treatment with $6\,mg\,kg^{-1}$ melphalan, on survival of KHT tumour cells. The numbers of tumours used at each dose level are indicated, together with the standard errors of the pooled survival determinations.

**Figure 3** The effect on tumour cell survival of giving hydralazine ($5\,mg\,kg^{-1}$) i.v. to mice at varying times before or after melphalan ($6\,mg\,kg^{-1}$). Symbols ±s.e. are surviving fractions derived from separate determinations on at least three tumours.

ing such a repair process, tumours from treated mice were excised 6 h after treatment and tumour cell survival compared with that obtained when excision was at 24 h. Results given in Table I show that hydralazine induced enhancement of cell killing is no different at 6 h from that at 24 h. This suggests that inhibition of repair processes is unlikely to contribute to the observed effect.

All the experiments so far described were carried out with subcutaneous tumours. Substantial differences in blood flow may occur in tumours implanted in alternative sites. Therefore, the effect of hydralazine on the toxicity of melphalan was evaluated in mice with the KHT tumour implanted into the gastrocnemius muscle. Table I shows that, for KHT cells implanted i.m., hydralazine also causes an increase in cell killing, similar to that observed for the subcutaneous tumour cells.

The effect of prolonged exposure to hydralazine was investigated. Table II lists values of surviving fraction from experiments where mice are administered $4\,mg\,kg^{-1}$ melphalan followed by a single or multiple doses of hydralazine given at 90 min intervals. Multiple doses of hydralazine alone have no cytotoxic effect on the KHT tumour. However, when combined with melphalan, there is an even greater enhancement of cell kill compared to that obtained following a single dose of hydralazine.

Growth delay of the KHT tumour has been used as an alternative assay of response following treatment of mice with melphalan or melphalan plus hydralazine. Growth curves were constructed for individual tumours and the data used to derive the time taken to reach 4× the initial treatment volume. Figure 4 shows mean values of growth delay for treated tumours (T) relative to untreated controls (Tc); plotted as a function of melphalan dose. Clearly, hydralazine when combined with melphalan, causes an increase in growth delay compared to that for melphalan alone. An ER of 3.0 was derived from linear regression lines fitted through the origin at zero melphalan dose. The value of ER derived using the growth delay data is close to that calculated from the results obtained by the clonogenic assay *in vitro*. Treatments wth hydralazine alone has no effect on growth of KHT tumours.

### *Therapeutic gain*

The systemic toxicity of treatment with the drug combination was compared to that for melphalan alone. This was

**Table I** Anti-tumour effect of various physical and chemical treatments when given 15 min after $6\,mg\,kg^{-1}$ melphalan to C3H mice carrying the KHT sarcoma

| *Treatment* | *Surviving fraction*[a] ($\times 10^2$) | *Number of tumours* |
|---|---|---|
| Melphalan alone | 9.6 (10.1–9.2) | 19 |
| Melphalan plus $5\,mg\,kg^{-1}$ hydralazine | 1.0 (1.2–0.83) | 11 |
| Melphalan plus clamp for 60 min | 0.82 (1.3–0.53) | 4 |
| Melphalan plus BW12C | 9.5 (9.8–9.2) | 6 |
| Excision at 6 h: | | |
| Melphalan alone | 8.5 (9.3–7.8) | 6 |
| Melphalan plus $5\,mg\,kg^{-1}$ hydralazine | 1.7 (2.1–1.4) | 6 |
| Intra-muscular tumours: | | |
| Melphalan alone | 17 (23–13) | 4 |
| Melphalan plus $5\,mg\,kg^{-1}$ hydralazine | 1.6 (4.0–0.64) | 4 |

[a]Numbers in parentheses indicate standard error limits.

**Table II** Effect of hydralazine on the cytotoxic action of 4 mg kg$^{-1}$ melphalan towards the KHT sarcoma

| *Treatment* | *Surviving fraction*[a] ($\times 10^2$) | *Number of tumours* |
|---|---|---|
| Melphalan alone | 27 (34–22) | 7 |
| Melphalan plus 5 mg kg$^{-1}$ hydralazine 15 min later: | 3.3 (3.7–2.9) | 10 |
| As above but in addition 3 subsequent doses of 5 mg kg$^{-1}$ hydralazine at 90 min intervals | 0.47 (0.56–0.41) | 5 |

[a]Numbers in parentheses indicate standard error limits.

**Figure 4** The effect of hydralazine (5 mg kg$^{-1}$) given i.v. to mice 15 min after treatment with varying doses of melphalan on growth of the KHT tumour. Animals were treated when tumours attained a mean diameter of about 6 mm and 6 to 8 animals were used per group. Values of T–Tc are given ± 1 s.e.

**Table III** $LD_{50}$ values for non-tumour bearing male C3H/He mice given melphalan with or without 5 mg kg$^{-1}$ hydralazine 15 min later

| | *Melphalan $LD_{50}$/mg kg$^{-1}$* | | |
|---|---|---|---|
| *Time* | *+ Hydralazine* | *− Hydralazine* | *ER* |
| 7 days | 16.8 | 19.5 | 1.16 |
| 90 days | 12.0 | 13.6 | 1.13 |

carried out in order to investigate whether any grounds existed for an increase in therapeutic benefit using this combined drug treatment. Lethal doses (LD50) were measured at either 7 or 90 days. These times were chosen to reflect relative toxicities in dose-limiting tissues i.e. gut and bone marrow respectively. Table III shows that there is a *small* increase in toxicity with the drug combination. However, the enhancement is substantially less than the ER observed for the tumour response.

### *Binding of $^3$H-misonidazole*

It is known that the radiosensitizer and bioreductive drug, misonidazole, is bound *in vivo* to oxygen deficient tissue (Garrecht & Chapman, 1983). Binding studies with isotopically labelled misonidazole offer an indirect method for determining the influence of hydralazine on blood flow, which will reflect itself as a change in oxygenation, in various tissues. The reductive process(es) leading to binding are inhibited by oxygen, therefore the efficiency of binding is a measure of the degree of hypoxia (van Os-Corby & Chapman, 1986). Mice were treated with tritiated-misonidazole with and without hydralazine as described previously and measurements of binding of label to various

**Figure 5** Binding of $^3$H-misonidazole to various tissues in C3H mice bearing the KHT tumour. A, misonidazole alone; B, misonidazole followed by hydralazine 15 min later; C, as for B but with two additional doses of hydralazine given at 90 min intervals thereafter.

**Table IV** Binding of $^3$H-misonidazole to tissues of mice bearing the KHT sarcoma: Ratio of level of binding; MISO + hydralazine relative to MISO alone

| *Treatment* | *Blood* | *Cerebelum* | *Spleen* | *Liver* | *Tumour* |
|---|---|---|---|---|---|
| MISO + hydralazine | 1.06 | 1.18 | 1.15 | 1.29 | 1.68[a] |
| MISO + 3 × hydralazine | 1.02 | 1.19 | 0.97 | 1.23 | 2.80[a] |

Hydralazine at 5 mg kg$^{-1}$ given i.v. 15 min post misonidazole and at 90 min intervals thereafter.

[a]Significantly different from a ratio of 1.0 at $P < 10^{-4}$.

mouse tissues are given in the histogram in Figure 5. In all treatment groups, significantly greater binding of labelled material was observed in the KHT tumour, the liver and the spleen compared to that in blood or cerebellum. For the group treated with misonidazole alone there is little difference between the tumour, liver and spleen. However, administration of single or multiple doses of hydralazine increases the amount of label in the tumour but not in any of the other tissues (Table IV). Further, the ratios of bound, labelled materal in tumour relative to blood give values of 1.9 in the group treated with misonidazole alone, 2.9 for the single dose of hydralazine and 4.4 in the group receiving three doses of hydralazine. Clearly, hydralazine substantially increases the efficiency of misonidazole binding and this appears to be tumour selective.

### Discussion

The major findings in this study are as follows:

1. Hydralazine substantially increases the anti-tumour effect of melphalan in murine KHT tumours. The use of two methods for measuring tumour response *in vivo*, clonogenic assay of cell survival *in vitro* and determination of tumour growth delay, both give similar values for enhancement ratios.
2. The enhancing effect of hydralazine shows a significant degree of tumour selectivity. The potentiation of the antitumour effect of melphalan is not reflected by a similar increase in either early or late systemic toxicity of melphalan. Some tumour selectivity is also evident in the enhancement of misonidazole-binding following treatment with hydralazine (Table IV).
3. Hydralazine, occlusion of the blood supply and treatment with BW12C all greatly increase the degree of hypoxia in the KHT tumour. However, BW12C does *not* enhance the anti-tumour action of melphalan, strongly suggesting that the degree of tumour hypoxia is *not* a factor in tumour sensitivity to melphalan.

Hydralazine is used in the treatment of acute hypertension. It acts mainly on the vascular smooth muscle causing peripheral vaso-dilation and decreased arterial blood pressure. It is known that hydralazine *reduces* but does not completely occlude blood flow in some experimental tumours (Cater *et al.*, 1962; Kruuv *et al.*, 1967; Vorhees & Babbs, 1982; Chan *et al.*, 1984; Chaplin & Acker, 1987). The data in Figure 3 show that hydralazine is effective in causing enhancement even when given *before* treatment with melphalan. This could be explained on the basis that hydralazine does not prevent the access of melphalan in the tumours but reduces the rate of tumour clearance. This would increase therefore the overall exposure of tumour cells to melphalan. A minor but possibly significant factor that may contribute to the enhancing effect of hydralazine may be a reduction in tumour pH caused by the reduced blood flow, increased hypoxia and retention of acid products of anaerobic metabolism. A small decrease in extracellular pH could increase the stability of melphalan (Ross, 1962) thereby leading to increased effectiveness of this drug.

Several studies have shown enhancement of the anti-tumour effect of melphalen *in vivo* by a variety of agents. Among these misonidazole, benznidazole and other nitroimidazoles can act as 'chemopotentiators'. The mechanism of the effect has its basis in bioreductive activation of the nitro compound causing damage that is only expressed when cells are exposed to a second cytotoxic treatment (Siemann, 1984). Changes in melphalan pharmacokinetics is also thought to be important (Lee & Workman, 1986) as well as changes in tumour vascular function (Murray *et al.*, 1987). The anaesthetic, safan, can increase the effectiveness of melphalan in the B16 melanoma but this is accompanied by an equivalent increase in normal tissue toxicity (Peacock & Stephens, 1978). Similarly, the vaso-dilating agent, verapamil, also increases the cytotoxicity of melphalan in some experimental tumours and some normal tissues (Robinson *et al.*, 1985). In this latter study it was shown that the effect of verapamil is to change melphalan pharmacokinetics and cellular uptake rather than causing any alterations in tumour blood flow (Robinson *et al.*, 1985, 1986).

The evidence from the work reported here indicates that hydralazine has a high degree of specificity in enhancing the anti-tumour effect of melphalan. If hydralazine, or other blood flow modifiers do not increase melphalan toxicity in dose-limiting human normal tissues, they could have a rôle to play in cancer chemotherapy, in addition to any use in combination with bioreductive radiosensitizing drugs (Chaplin & Acker, 1987; Brown 1987; Stratford *et al.*, 1987).

### References

ADAMS, G.E., BARNES, D., LOUTIT, J. & 9 others (1986). Induction of hypoxia in normal and malignant tissues by changing the oxygen affinity of haemoglobin: Implications for therapy. *Int. J. Radiat. Oncol. Biol. Phys.*, **12**, 1299.

ALGIRE, G.H. & LAGALLAIS, F.Y. (1951). Vascular reactions of normal and malignant tissues *in vivo* IV. The effect of peripheral hypotension on transplanted tumours. *J. Natl Cancer. Inst.*, **12**, 399.

BORN, J.L. & SMITH, B.R. (1983). The synthesis of tritium labelled misonidazole. *J. Labelled Compounds Radiopharmaceut.*, **20**, 429.

BROWN, J.M. (1987). Exploitation of bioreductive agents with vasoactive drugs. In *Radiation Research* Vol. 2. Fielden, *et al.* (eds) p.719. Taylor & Francis: London.

CATER, D.B., GRIGSON, C.M.B. & WATKINSON D.A. (1962). Changes of oxygen tension induced by vaso-constrictor and vasodilator drugs. *Acta Radiol.*, **58**, 401.

CHAN, R.C., BABBS, C.F., VETTER, R.J. & LAMAR, C.H. (1984). Abnormal response of tumour vasculature to vasoactive drugs. *J. Natl Cancer. Inst.*, **72**, 145.

CHAPLIN, D.J. & ACKER, B. (1987). Potentiation of RSU 1069 tumour cytotoxicity by hydralazine: A new approach to selective therapy. *Int. J. Radiat. Oncol. Biol. Phys.*, **13**, 579.

COURTENAY, D.V. (1983). The Courtenay clonogenic assay. In *Human Tumour Drug Sensitivity Testing in vitro: Techniques and Clinical Applications*, Dendy, P.P. & Hill, B.T. (eds) p. 101. Academic Press: London.

GARRECHT, B.M. & CHAPMAN, J.D. (1983). The labelling of EMT-6 tumours with $^{14}$C-misonidazole. *Br. J. Radiol.*, **56**, 745.

KALLMAN, R.F., SILINI, G. & VAN PUTTEN, L.M. (1967). Factors influencing the quantitative estimation of the *in vivo* survival of cells from solid tumours. *J. Natl Cancer Inst.*, **39**, 539.

KNAPP, W.H., DEBATIN, J., LAYER, K. & 4 others (1985). Selective drug induced reduction of blood flow in tumour transplants. *Int. J. Radiat. Oncol. Biol. Phys.*, **11**, 1357.

KRUUV, J.A., INCH, W.R. & MCCREDIE, J.A. (1967). Blood flow and oxygenation of tumours in mice II. Effects of vasodilators. *Cancer*, **20**, 60.

LEE, F.Y.F. & WORKMAN, P. (1986). Altered pharmacokinetics in the mechanism of chemosensitization – effects of nitroimidazoles and other chemical modifiers on the pharmacokinetics, anti-tumour activity and acute toxicity of selected nitrogen mustards. *Cancer Chemother. Pharmacol.*, **17**, 30.

LITTLE, J.B., HAHN, G.M., FRINDEL, E. & TUBIANA, M. (1973). Repair of potentially lethal radiation damage *in vitro* and *in vivo*. *Radiology*, **106**, 689.

McNALLY, N.J. & SHELDON, P.W. (1977). The effect of radiation tumour growth delay, cell survival and cure of the animal using a single tumour system. *Br. J. Radiol.*, **50**, 321.

MURRAY, J.C., RANDHAWA, V. & DENEKAMP, J. (1987). The effects of melphalan and misonidazole on the vasculature of a murine sarcoma. *Br. J. Cancer*, **55**, 233.

VAN OS-CORBY, D.J. & CHAPMAN, J.D. (1986). *In vitro* binding of $^{14}$C-misonidazole to hepatocytes and hepatoma cells. *Int. J. Radiat. Oncol. Biol. Phys.*, **12**, 1251.

PEACOCK, J.H. & STEPHENS, T.C. (1978). Influence of anaesthetics on tumour cell kill and repopulation in B16 melanoma treated with melphalan. *Br. J. Cancer*, **38**, 725.

ROBINSON, B.A., CLUTTERBUCK, R.D., MILLAR, J.L.. & McELWAIN, T.J. (1985). Verapamil potentiation of melphalan cytotoxicity and cellular uptake in murine fibrosarcoma and bone marrow. *Br. J. Cancer*, **52**, 813.

ROBINSON, B.A., CLUTTERBUCK, R.D., MILLAR, J.L. & McELWAIN, T.J. (1986). Effects of verapamil and alcohol on blood flow, melphalan uptake and cytotoxicity in murine fibrosarcomas and human melanoma xenografts. *Br. J. Cancer*, **53**, 607.

ROSS, W.C.J. (1962). *Biological Alkylating Agents*. Butterworths: London, p. 99.

SIEMANN, D.W. (1984). Modification of chemotherapy by nitroimidazoles. *Int. J. Radiat. Oncol. Biol. Phys.*, **10**, 1585.

STRATFORD, I.J., ADAMS, G.E., GODDEN, J. & HOWELLS, N. (1988). Induction of tumour hypoxia post-irradiation: A method for increasing the sensitizing efficiency of misonidazole and RSU 1069 *in vivo*. *Int. J. Radiat. Biol.*, (in press).

STRATFORD, I.J., GODDEN, J., HOWELLS, N., EMBLING, P. & ADAMS, G.E. (1987). Manipulation of tumour oxygenation by hydralazine increases the potency of bioreductive radiosensitizers and enhances the effect of melphalan in experimental tumours. *Radiation Research*, **Vol. 2**, Fielden, *et al.* (eds) p. 737. Taylor & Francis: London.

STRATFORD, I.J., WALLING, J.M. & SILVER, A.R.J. (1986). The differential cytotoxicity of RSU 1069: Cell survival studies indicating interaction with DNA as a possible mode of action. *Br. J. Cancer*, **53**, 339.

THOMSON, J.E. & RAUTH, A.M. (1974). An *in vitro* assay to measure the viability of KHT tumour cells not previously exposed to culture conditions. *Radiat. Res.*, **58**, 262.

VOORHEES, W.D. & BABBS, C.F. (1982). Hydralazine-enhanced selective heating of transmissible venereal tumour implants in dogs. *Eur. J. Cancer Clin. Oncol.*, **19**, 1027.